# Observational Study Designs: Synopsis for Selecting an Appropriate Study Design

**DOI:** 10.7759/cureus.6692

**Published:** 2020-01-17

**Authors:** Assad A Rezigalla

**Affiliations:** 1 Department of Basic Medical Sciences, College of Medicine, University of Bisha, Bisha, SAU

**Keywords:** observational, study design, descriptive, retrospective, prospective, strength

## Abstract

The selection of a study design is the most critical step in the research methodology. Crucial factors should be considered during the selection of the study design, which is the formulated research question, as well as the method of participant selection. Different study designs can be applied to the same research question(s). Research designs are classified as qualitative, quantitative, and mixed design. Observational design occupies the middle and lower parts of the hierarchy of evidence-based pyramid. The observational design is subdivided into descriptive, including cross-sectional, case report or case series, and correlational, and analytic which includes cross-section, case-control, and cohort studies. Each research design has its uses and points of strength and limitations. The aim of this article to provide a simplified approach for the selection of descriptive study design.

## Introduction and background

**Figure 1:**
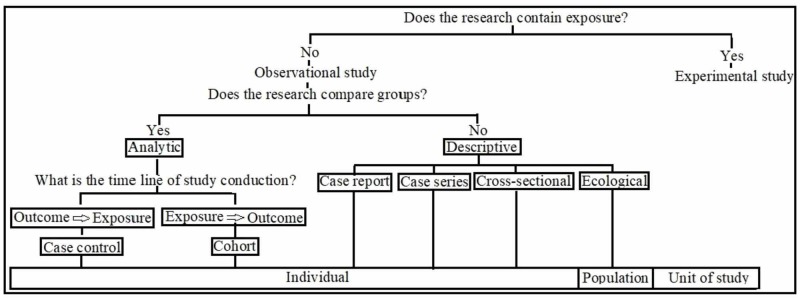
Classification of observational study design.

A research design is defined as the “set up to decide on, among other issues, how to collect further data, analyze and interpret them, and finally, to provide an answer to the question” [1]. The primary objective of a research design is to guarantee that the collected evidence allows the answering of the initial question(s) as clearly as possible [2]. Various study designs have been described in the literature [1-3]. Each of them deals with the specific type of research or research questions and has points of strength and weakness. Broadly, research designs are classified into qualitative and quantitative research and mixed methods [3]. The quantitative study design is subdivided into descriptive versus analytical study designs or as observational versus interventional (Figure 1). Descriptive designs occupy the middle and lower parts of the hierarchy of evidence-based medicine pyramid. Study designs are organized in a hierarchy beginning from the basic "case report" to the highly valued "randomised clinical trial" [4-5].

## Review

Case report

The case report describes an individual case or cases in their natural settings. Also, it describes unrecognized syndromes or variants, abnormal findings or outcomes, or association between risk factors and disease. It is the lowest level and the first line of evidence and usually deals with the newly emerging issues and ideas (Table 1) [4, 6-10].

**Table 1:** Strengths and Limitations of Case Report Design

Case Report Design	
Strengths [4, 6-9]	Limitations [6, 9]
Identification of new, abnormal, or variant presentation of diseases.	Lack of generalizability and implications.
Have significant educational value.	Uncontrolled.
Help in generating a hypothesis.	Selection bias.
Researching rare or uncommon disorders.	No epidemiological indices (parameters).
In-depth narrative case studies.	Over-interpretation.
Flexible structure.	Confidentiality.
	Causes may have other explanations.

Case series

A case series is a report on data from a subject group (multiple patients) without control [6, 11-12]. Commonly, this design is used for the illustration of novel, unusual, or atypical features identified in medical practice [6]. The investigator is governed by the availability and accuracy of the records, which can cause biases [13-14]. Bias in a case series can be decreased through consecutive patient enrollment and predefined inclusion and exclusion criteria, explicit specification of study duration, and enrollment of participants (Table 2) [11-12].

**Table 2:** Strengths and Limitations of Case Series Design

Case Series	
Strengths [6, 11-12]	Limitations [6, 11-12]
Educational.	Selection bias.
It described the outcomes of novel treatments.	Lack of control.
The gained information can be used to generate hypotheses.	Difficult to compare different cases.
Provide strong evidence with multiple cases.	The result may not be generalized.
Helpful in refining new techniques or treatment protocols.	Immediate follow-up.
Identify the rare manifestations of a disease or drug.	Have a lower position on the hierarchy of evidence.
Feasible study designs.	

Correlational study design

Correlational studies (ecologic studies) explore the statistical relationships between the outcome of interest in population and estimate the exposures. It deals with the community rather than in individual cases. The correlational study design can compare two or more relevant variables and reports the association between them without controlling the variables. The aim of correlational study design or research is to uncover any types of systematic relationships between the studied variables. Ecological studies are often used to measure the prevalence and incidence of disease, mainly when the disease is rare. The populations compared can be defined in several ways, such as geographical, time trends, migrants, longitudinal, occupation, and social class. It should be considered that in ecological studies, the results are presented at the population (group) level rather than individuals. Ecological studies do not provide information about the degree or extent of exposure or outcome of interest for particular individuals within the study group (Table 3) [7, 15-16]. For example, we do not know whether those individuals who died in the study group under observation had higher exposure than those remained alive.

**Table 3:** Strengths and Limitations of Correlation Study Design

Correlational study design	
Strengths [15-16]	Limitations [15-16]
Quick and easy.	Correlations do not equal causation.
Describes the strength of relationships.	Correlations can be misused.
It is used to assess behavior.	Cannot be used to identify causal relationships
Predictor variables cannot be manipulated.	It cannot provide certain information.
Uses of data records.	

Cross-sectional study design

The cross-sectional study examines the association between exposures and outcomes on a snap of time. The assessed associations are guided by sound hypotheses and seen as hypothesis-generating [17]. This design can be descriptive (when dealing with prevalence or survey) or analytic (when comparing groups) [17-18]. The selection of participants in a cross-sectional study design depends on the predefined inclusion and exclusion criteria [18-19]. This method of selection limits randomization (Table 4).

**Table 4:** Strengths and Limitations of Cross-sectional Study Design

Cross-sectional Study Design	
Strengths of [17, 19-20]	Limitations [17, 19-20]
Fast and inexpensive.	Difficult to derive causal relationships.
Useful for planning monitoring and evaluation of public health.	Prone to certain types of biases.
Efficient in studying rare diseases.	The response rate is critical.
There are seldom ethical difficulties.	The temporality of the design.
It can assess multiple outcomes.	No clear demarcation between exposure and effect.
Population-based surveys.	
Estimation of prevalence.	
Calculation of odds ratio.	
The baseline for a cohort study.	

Case-control study

A case-control study is an observational analytic retrospective study design [12]. It starts with the outcome of interest (referred to as cases) and looks back in time for exposures that likely caused the outcome of interest [13, 20]. This design compares two groups of participants - those with the outcome of interest and the matched control [12]. The controls should match the group of interest in most of the aspects, except for the outcome of interest [18]. The controls should be selected from the same localization or setting of the cases [13, 21-22]. Case-control studies can determine the relative importance of a predictor variable about the presence or absence of the disease (Table 5).

**Table 5:** Strengths and Limitations of Case-control Study Design

Case-control Study Design	
Strengths [12, 20-21]	Limitations [12, 20-21]
Relatively fast in conduction in comparison with prospective cohort studies.	Not useful for rare exposures.
Comparatively, needs few participants and fewer resources.	Cannot estimate the incidence.
Useful for testing hypotheses.	Affect by observation and recall bias.
Useful in studying multiple exposures in the same outcome.	
Can study the association of risk factors and outcomes in outbreak investigations.	
It can generate much information from relatively few participants with unusual cases.	
Feasible in diseases with a long latent period.	

Cohort study design

The cohort study design is classified as an observational analytic study design. This design compares two groups, with exposure of interest and control one [12, 18, 22-24].

Cohort design starts with exposure of interest comparing them to non-exposed participants at the time of study initiation [18, 22, 24]. The non-exposed serve as external control. A cohort design can be either prospective [18] or retrospective [12, 20, 24-25]. In prospective cohort studies, the investigator measures a variety of variables that might be a risk factor or relevant to the development of the outcome of interest. Over time, the participants are observed to detect whether they develop the outcome of interest or not. In this case, the participants who do not develop the outcome of interest can act as internal controls. Retrospective cohort studies use data records that were documented for other purposes. The study duration may vary according to the commencement of data recording. Completion of the study is limited to the analysis of the data [18, 22, 24]. In 2016, Setia reported that, in some instances, cohort design could not be well-defined as prospective or retrospective; this happened when retrospective and prospective data were collected from the same participants (Table 6) [24].

**Table 6:** Strengths and Limitations of Cohort Study Design

Cohort Study Design	
Strengths [12, 20, 24]	Limitations [12, 20, 24]
The temporality between exposure and outcome is well-defined.	Inability to control all the confounding variables.
Study multiple outcomes in the same exposure.	A prospective cohort design is time-consuming and costly.
Efficient in rare outcomes if the rare outcome is common in some exposures.	Variables in the retrospective cohort study may not be very accurate since the collected data was not intended for research purposes.
Accurate measure of variables in prospective cohort design.	May not be very useful in case of rare outcomes.
The retrospective cohort is relatively fast in conduction and inexpensive.	In the prospective cohort design, the loss of follow-up is a critical problem.
Lack of bias in the retrospective cohort because the collected data was not initially for research.	Retrospective cohorts may be affected by recall bias.
It can measure potential causes and relative risk.	Ethical problems.

The selection of the study design is the most critical step in research methodology [4, 26]. An appropriate study design guarantees the achievement of the research objectives. The crucial factors that should be considered in the selection of the study design are the formulated research question, as well as the method of sampling [4, 27]. The study design determines the way of sampling and data analysis [4]. The selection of a research study design depends on many factors. Two crucial points that should be noted during the process selection include different study designs that may be applicable for the same research question(s) and researches may have grey areas in which they have different views about the type of study design [4].

## Conclusions

The selection of appropriate study designs for research is critical. Many research designs can apply to the same research. Appropriate selection guarantees that the author will achieve the research objectives and address the research questions.
